# Recurrence of Small Bowel Obstruction in Adults After Operative Management of Adhesive Small Bowel Obstruction: A Systematic Review

**DOI:** 10.7759/cureus.29141

**Published:** 2022-09-13

**Authors:** Nishok Victory Srinivasan, Aujala Irfan Khan, Ghadi D Mashat, Mohammad Hazique, Kokab Irfan Khan, Prasana Ramesh, Suthasenthuran Kanagalingam, FNU Zargham Ul Haq, Sai Sri Penumetcha

**Affiliations:** 1 General Surgery, California Institute of Behavioral Neurosciences & Psychology, Fairfield, USA; 2 Research, California Institute of Behavioral Neurosciences & Psychology, Fairfield, USA; 3 Pediatrics, California Institute of Behavioral Neurosciences & Psychology, Fairfield, USA; 4 Internal Medicine, California Institute of Behavioral Neurosciences & Psychology, Fairfield, USA; 5 Internal Medicine/Family Medicine, California Institute of Behavioral Neurosciences & Psychology, Fairfield, USA; 6 Medicine and Surgery, California Institute of Behavioral Neurosciences & Psychology, Fairfield, USA; 7 Internal Medicine, Chalmeda Anand Rao Institute of Medical Sciences, Karimnagar, India, IND

**Keywords:** adhesive small bowel obstruction, recurrent small bowel obstruction, conservative and surgical treatment, open and laparoscopic surgery, adhesiolysis, small-bowel obstruction

## Abstract

The objective of this article is to review the existing literature on postoperative recurrence of adhesive small bowel obstruction (ASBO). We performed a systematic review following Preferred Reporting Items for Systematic Reviews and Meta-Analyses (PRISMA) guidelines, searching PubMed, Cochrane Library, and Google Scholar, to identify randomized controlled trials (RCTs) and observational studies investigating recurrence after operative management of ASBO. Our search yielded one RCT, one prospective study, and eight retrospective studies, totaling 36,178 patients. We used Cochrane risk-of-bias tool and the Newcastle-Ottawa scale to assess the risk of bias in the reviewed studies (RCTs and observational studies, respectively). Operative management was associated with a lower risk of recurrence than conservative management, while the difference in recurrence between laparoscopic and open surgery was inconclusive. Diffuse adhesions were associated with a greater risk of recurrence than single band adhesions. We conclude that the “common knowledge” that surgery increases the risk for recurrence of ASBO is outdated and should no longer be applied when determining treatment modalities for ASBO. While conservative treatment still has its place, we need not fear the possibility of shifting patients to operative management earlier.

## Introduction and background

Small bowel obstruction (SBO) is a common problem with various etiologies. Of those, adhesion-related obstructions are the most common, accounting for 15% (an estimated 300,000 admissions per year) of emergent SBO admissions in North America and 74% of all SBO admissions [1,2]. Around 93-100% of patients who undergo intra-abdominal surgery will develop adhesions [3], and approximately 80% of all adhesive SBO (ASBO) patients have a history of previous intra-abdominal surgery [4].

ASBO may be treated by non-operative (conservative) measures or open/laparoscopic surgery (lysis of adhesions). Non-operative treatment involves bowel rest with fluid resuscitation, bowel decompression (with a nasogastric tube), and correcting electrolyte abnormalities while waiting for the passage of flatus or stools (return of bowel function) [3]. More recently, administration of gastrografin orally or via nasogastric tube has been suggested to stimulate the bowel and hasten the return of bowel function [5,6]. Operative management involves open or laparoscopic lysis of the offending adhesions, relieving the obstruction.

Non-operative management is successful in about 80% of cases [7]. However, it leaves the offending adhesions in place, risking the recurrence of the issue. On the other hand, surgical intervention is a more definitive treatment but comes with the risk of forming new adhesions [3,8]. With both treatment options involving a risk of recurrence, we must ask ourselves which option is better in terms of overall outcomes.

Despite the ongoing debate, there is a scarcity of existing literature that focuses specifically on the risk of recurrence of adhesive SBO after treatment. Therefore, this review aims to evaluate the risk of recurrence of SBO in patients undergoing operative management of ASBO.

## Review

Methods

We reviewed the literature according to Preferred Reporting Items for Systematic Reviews and Meta-Analyses (PRISMA) [9].

Eligibility Criteria

All observational studies and randomized controlled trials (RCTs) detailing the recurrence of short bowel obstruction after operative management of adhesive short bowel obstruction (SBO) were included. The study population consisted of adult (age > 18 years) humans. Studies published in English with a minimum of 100 patients that underwent operative management for SBO were included. A minimum median follow-up period of one year was required for inclusion.

Other causes of SBO, such as malignancy, Crohn’s disease, hernia, volvulus, and sclerosing encapsulating peritonitis, were excluded from this study. Case reports and existing traditional/systematic reviews were also excluded.

Outcomes

Recurrence of SBO was considered the primary outcome. Time to recurrence and treatment of recurrent SBO were the secondary outcomes.

Literature Search Strategy and Study Selection

The first and second authors searched the following databases on June 8, 2022: PubMed, Cochrane Library, and Google Scholar. The complete search strategy is included in Table 1. The investigation was limited to studies published from 2000 to 2022, in the English language, with an adult study population.

**Table 1:** Search strategies MeSH, medical subject headings

Database	Search Strategy	Filters
PubMed	(relapse OR post-surgery recurrence OR recurrence OR post-op recurrence OR post-operative recurrence OR "Recurrence/surgery"[Majr]) AND (Intestinal obstruction OR short bowel obstruction OR small bowel obstruction OR small intestine obstruction OR ("Intestinal Obstruction/surgery"[Majr] OR "Intestinal Obstruction/therapy"[Majr]))	Years 2000-2022, adult, human, English
Cochrane Library	(MeSH descriptor: [Intestinal obstruction] explode all trees) AND (MeSH descriptor: [Recurrence] explode all trees OR post-op recurrence OR post-surgery recurrence OR recurrence OR relapse)	Years 2000-2022
Google Scholar	("intestinal obstruction" OR "short bowel obstruction" OR "short intestine obstruction") AND ("recurrence" OR "post-op recurrence" OR "post-surgery recurrence" OR relapse)	Years 2000-2022, English

**Figure 1:**
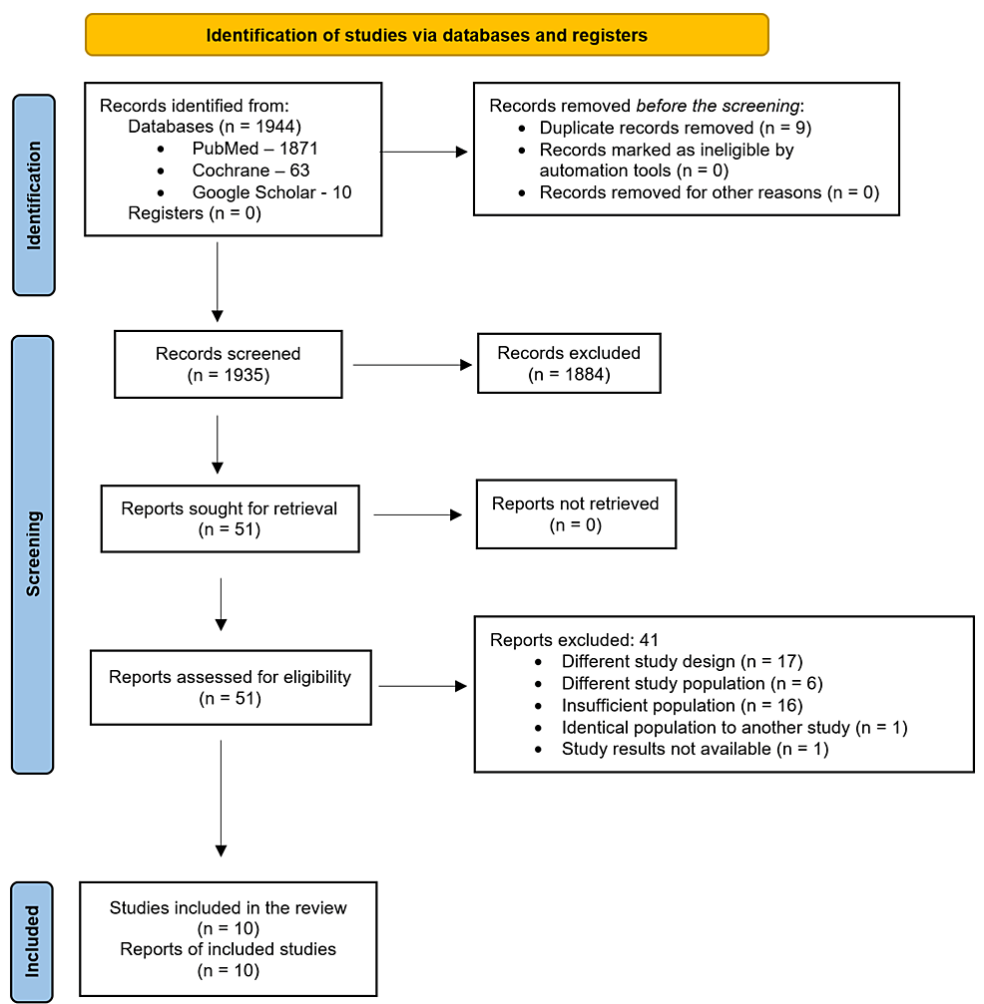
PRISMA flowchart PRISMA - Preferred Reporting Items for Systematic Reviews and Meta-Analyses Cochrane - Cochrane Library

Articles from the literature search were screened by the titles and abstracts. Shortlisted studies had their full text retrieved, and those that met the inclusion criteria were selected for the review, as detailed in the PRISMA flowchart below (Figure 1).

Quality Assessment

We assessed the risk of bias in RCTs with the Cochrane risk-of-bias tool (Rob-2). The quality of observational studies was assessed using the Newcastle-Ottawa scale (NOS). The Cochrane tool classifies studies into low, unclear, and high risk of bias. The NOS classifies studies into low, moderate, or high risk of bias (nine stars, seven or eight stars, and six or fewer stars, respectively).

Results

Search Results

From our literature search of 1,956 articles, we shortlisted 51 studies for further perusal. After obtaining the full texts, we found 10 studies eligible for the review - one RCT, one prospective study, and eight retrospective observational studies - enrolling 36,178 patients (19,021 female). Of those, 9,878 underwent operative management (open or laparoscopic adhesiolysis, with or without small bowel resection). Fourteen patients recruited had non-adhesive causes of SBO (one study [10] did not specify the underlying causes of SBO in the study population). A total of 185 patients did not have a history of abdominal surgery before the incidence of SBO (three studies [8,11,12] did not record prior surgical history).

The characteristics of included studies and the study populations are shown in Table 2 and Table 3, respectively. The primary and secondary outcomes are detailed in Table 4 and Table 5, respectively.

**Table 2:** Study characteristics ASBO, adhesive small bowel obstruction; SBO, small bowel obstruction; IQR, inter-quartile range; ICD, International Classification of Diseases; ICD-10 K56.5, code for "Intestinal adhesions (bands) with obstructions"; JAMA, Journal of the American Medical Association; BMC, BioMed Central Ltd.

Study	Journal	Study design	Patient presentation	Comparison	Follow-up period (median years)
Duron et al. [13]	Annals of Surgery	Prospective study	Postoperative ASBO undergoing operative management	None	3.4 (range: 0.08-6.25)
Catena et al. [11]	Journal of Gastrointestinal Surgery	Randomized controlled trial	ASBO with surgical indication to laparotomy	Traditional treatment with intraperitoneal icodextrin 4% vs. traditional treatment (control)	3.45
Meier et al. [10]	World Journal of Surgery	Retrospective observational	Acute SBO - Intestinal adhesions with obstruction (ICD-10 K56.5)	Operative management group vs. non-operative management group	4.7 (IQR 3.7-5.8)
Nakamura et al. [14]	Surgical Laparoscopy Endoscopy & Percutaneous Techniques	Retrospective observational	Adhesive postoperative SBO undergoing operative management	Open surgery vs. laparoscopic surgery	4.75 (range: 0.58-15.4)
Yao et al. [15]	Surgical Endoscopy	Retrospective observational	SBO undergoing adhesiolysis	Open surgery vs. laparoscopic surgery	3.92 (range: 0-8.83)
Lorentzen et al. [16]	Journal of Gastrointestinal Surgery	Retrospective observational	Emergent surgery for ASBO	Without recurrent ASBO vs. recurrent ASBO	2.2 (range: 0-10.6)
Mu et al. [17]	Medicine	Retrospective observational	ASBO	Operative management group vs. non-operative management group	2 (0.5-3.1)
Behman et al. [8]	JAMA Surgery	Retrospective observational	The first episode of ASBO	Operative management group vs. non-operative management group	3.6 (IQR 1.4-6.5)
Medvecz et al. [12]	Journal of American College of Surgeons	Retrospective observational	Short bowel obstruction due to adhesive disease	Operative management group vs. non-operative management group	9.82/9.88 (operative/non-operative groups)
Sakari et al. [18]	BMC Surgery	Retrospective observational	ASBO undergoing operative management	Band adhesions vs. diffuse adhesions causing SBO (AND) age < 70 vs. age > 70 years	5.5 (range: 0-10.16)

**Table 3:** Population baseline characteristics SBO, short bowel obstruction

Paper	Sample size	Female	Adhesions causing SBO	Previous abdominal surgery
Duron et al. [13]	286	186	286	286
Catena et al. [11]	181	100	181	Not mentioned
Meier et al. [10]	221	131	Not mentioned	201
Nakamura et al. [14]	123	65	109	123
Lorentzen et al. [16]	478	298	478	418
Yao et al. [15]	104	53	104	92
Mu et al. [17]	288	137	288	245
Behman et al. [8]	27,904	14,228	27,904	Not mentioned
Medvecz et al. [12]	6,191	3,583	6,191	Not mentioned
Sakari et al. [18]	402	240	402	352

**Table 4:** Recurrence of SBO and time to first recurrence SBO, short/small bowel obstruction; Rx, treatment; NA, not available; p-value, probability value

Paper	Treatment group	Sample size	Recurrence (%)	p-Value	Median time to recurrence (months)	p-Value
Duron et al. [13]	Operative Rx	286	33 (12%)	NA	NA	NA
Lorentzen et al. [16]	Operative Rx	478	58 (12.1)	NA	6	NA
Meier et al. [10]	Operative Rx	136	19 (14)	0.014	13	0.121
	Non-operative Rx	85	25 (29.4)		20	
Mu et al. [17]	Operative Rx	122	26 (21.3)	0.01	19.3	NA
	Non-operative Rx	166	58 (34.9)		33.1	
Behman et al. [8]	Operative Rx	6,186	804 (13.0)	<0.01	NA	
	Non-operative Rx	21,718	4,626 (21.3)		NA	
Medvecz et al. [12]	Operative Rx	1,860	354 (19.0)	<0.005	24.3	0.009
	Non-operative Rx	4,331	1,112 (25.6)		18.3	
Nakamura et al. [14]	Open surgery	48	9 (18.75)	0.018	NA	NA
	Laparoscopic surgery	75	3 (4)		NA	
Yao et al. [15]	Open surgery	52	4 (7.7)	0.505	NA	NA
	Laparoscopic surgery	52	6 (11.5)		NA	
Sakari et al. [18]	Operative Rx for band adhesions	226	33 (15)	0.05	NA	NA
	Operative Rx for diffuse adhesions	176	39 (22)		NA	
Catena et al. [11]	Operative Rx with icodextrin	91	2 (2.2)	<0.05	NA	NA
	Operative Rx without icodextrin	90	10 (11.1)		NA	

**Table 5:** Treatment of recurrent SBO episodes SBO, short/small bowel obstruction; p-value, probability value; Rx, treatment; NA, not available

Paper	SBO treatment group	Recurrence	Recurrence treated conservatively (%)	Recurrence treated operatively (%)	p-Value
Duron et al. [13]	Operative Rx	33	22 (66.7)	11 (33.3)	NA
Lorentzen et al. [16]	Operative Rx	58	15 (26)	43 (74)	NA
Meier et al. [10]	Operative Rx	19	9 (47.4)	10 (52.6)	>0.05
	Non-operative Rx	25	19 (76)	6 (24)	
Nakamura et al. [14]	Open surgery	9	1 (11.1)	8 (88.9)	0.038
	Laparoscopic	3	0 (0)	3 (100)	
Yao et al. [15]	Open surgery	4	4 (100)	0 (0)	0.017
	Laparoscopic	6	2 (33.3)	4 (66.7)	
Catena et al. [11]	Operative Rx with icodextrin	2	1 (50)	1 (50)	>0.05
	Operative Rx without icodextrin	10	7 (70)	3 (30)	
Sakari et al. [18]	Operative Rx for band adhesions	33	20 (60.6)	13 (39.4)	0.509
	Operative Rx for diffuse adhesions	39	26 (66.7)	13 (33.3)	
Total	Operative Rx	216	107 (49.5)	109 (50.5)	
	Non-operative Rx	25	19 (76)	6 (24)	

Discussion

From our literature search of 1,956 articles, we shortlisted 51 studies for further perusal. After obtaining the full texts, we found 10 studies eligible for the review - one RCT, one prospective study, and eight retrospective observational studies - enrolling a total of 36,178 patients, with 7,221 episodes of recurrent SBO after treatment. The incidence of recurrent SBO ranged from as low as 2.2% to a maximum of 34.9% [11,17].

Two studies [13,16] only reported recurrence after operative treatment. Four studies [8,10,12,17] reported recurrence after operative and conservative treatment, comparing the two. The risk of recurrence after operative management ranged from 12% to 19%, significantly lower than the risk in the conservative management group. Behman et al. reported a relative risk reduction (RRR) of 41% with operative treatment over conservative [8]. Operative treatment allows for the takedown of adhesions but promotes the formation of new ones [3,8]. Conservative treatment does not form new adhesions but leaves the initial adhesions that caused the obstruction in place. Our review shows that contrary to popular belief, lysis of existing adhesions is less likely to cause recurrent SBO than conservative treatment despite the risk of forming new adhesions after surgery.

Nakamura et al. reported an increased risk of recurrence with laparotomy over laparoscopy, while Yao et al. had no significant difference [14,15]. Behman et al. also claimed that laparoscopy did not significantly decrease the hazard of recurrence associated with operative management [8]. Open surgical procedures are associated with increased adhesions compared to laparoscopy, which explains the increased risk of SBO recurrence after laparotomy in the study by Nakamura et al. However, Yao et al. theorize that laparoscopy results in incomplete adhesiolysis due to the limited field of vision and exposure of the small bowel, which could translate to an increased risk of recurrence from the adhesions left behind. The decisive obstruction may be relieved while overlooking occult points of obstruction [15]. This would mean that the surgeon’s skill plays a more significant role in recurrence after laparoscopy. Further research, particularly with RCTs, is necessary to determine whether laparoscopy has a lesser risk of recurrence than laparotomy for operative management of adhesive SBO. Meanwhile, using laparoscopy for general abdominal surgeries, proven to cause less adhesions than open surgery, may help prevent the index episode of adhesive SBO [19,20].

Sakari et al. concluded that the type of adhesions influenced recurrence - diffuse adhesions were associated with a higher recurrence rate than single band adhesions [18]. Lorentzen et al. and Duron et al. came to similar conclusions after multivariate analysis, multiple or matted adhesions were associated with an increased risk of recurrence [13,16]. Diffuse adhesions increase the difficulty of adhesiolysis, which translates to increased peritoneal trauma and inflammation, a risk factor for adhesion formation [13,18]. Difficult adhesiolysis also increases the risk of bowel injury, and diffuse adhesions are associated with a higher number of prior abdominal surgeries [18], increasing the chances of recurrence. Yao et al. also determined that a higher number of previous abdominal surgeries is a risk factor for recurrence [15].

On the other hand, Catena et al. tested the application of icodextrin, an anti-adhesion agent, after operative management of SBO in an RCT. They reported a significant decrease in recurrence in the icodextrin arm (2.2% vs. 11.1%) [11]. This may become the standard of care for operative management of adhesive SBO. We can also extrapolate its application to other abdominal surgeries to prevent the initial formation of adhesions that cause the index episode of SBO.

Four studies included the time interval between initial treatment and recurrence (hereafter referred to as “time to recurrence”) [10,12,16,17]. Medvecz et al. had the only statistically significant result, with a lower time to recurrence in patients who underwent operative treatment (compared to conservative therapy). Mu et al. and Meier et al. reported the opposite - operative treatment had a shorter time to recurrence - but the results were not statistically significant. Lorentzen et al. reported an oddly short time to recurrence (six months), far shorter than the other three studies. The reason for this is unknown.

According to Behman et al., with every consecutive episode of SBO treated conservatively, the risk of recurrence increases (19.2% after the first episode to 48% after the third episode). On the other hand, surgical intervention after any number of recurrences was associated with a significant decrease in subsequent recurrences (RRR of 51% compared to non-operative intervention) [8]. This is a continuation of the trend of operative management having a lower risk of recurrence than conservative management. Medvecz et al. also reported similar results, theorizing that this is due to a cycle - multiple episodes of recurrence increase the risk of adhesion formation, resulting in further recurrence, cycling back [12]. Operative treatment significantly decreases the risk of recurrence, breaking the cycle. This potential “hidden” benefit may counterbalance the inherent risks of surgery.

Nakamura et al. and Yao et al. also reported that patients who underwent laparoscopic treatment for the initial SBO episode are more likely to require operative management for recurrent episodes [14,15]. This likely reflected laparoscopy leading to incomplete adhesiolysis and missed occult obstructions, as discussed earlier. This is additional proof that laparoscopy might not be a promising treatment option for adhesive SBO, despite the lower morbidity and adhesion formation.

Duron et al. and Lorentzen et al. also reported that bowel resection was significantly associated with a decreased risk of recurrence. Duron et al. theorized that the underlying reason was two-fold - one, it removed a length of traumatized and inflamed serosa (from adhesiolysis), reducing the length of bowel available for adhesion formation, and, two, it reduced peritoneal inflammation by decreasing the need for lysis of adhesions [13]. Lorentzen et al. however, proposed that the reduced recurrence was because patients with single band adhesions were more likely to undergo bowel resection than those with diffuse adhesions [16,21]; the decreased recurrence was because patients with single band adhesions were inherently less likely to develop recurrence.

According to Duron et al., patients under 40 exhibited an increased risk for recurrence. This has not been reported in any other study, and the underlying process is unknown. Studies on infants have shown a higher degree of adhesion formation after abdominal surgery, but no such studies have been performed in young adults. Another theory by Duron et al. is that elderly patients may have a lower risk of adhesion formation due to decreased gastrointestinal motility [13]. Another odd data point was reported by Lorentzen et al. and Sakari et al., who reported that female patients had significantly higher recurrence rates than males. The underlying reasoning is unknown, but Sakari et al. proposed that this might stem from the incidence of gynecological surgeries in women [16,18].

Lorentzen et al. also reported an increased risk of recurrence if postoperative fascial dehiscence occurred. This is because fascial dehiscence requires reoperation, which means an increased risk for the formation of adhesions [16]. Mu et al. discovered that strangulating bowel obstruction is not an independent risk factor for recurrence despite requiring emergent surgery over elective surgery or conservative measures [17], another finding that supports operative management.

The overall trend of this review appears to show that operative treatment for ASBO is a viable option, with a lower chance of recurrence than conservative treatment. This is at odds with the current guidelines for SBO treatment, which prioritize conservative treatment for as long as possible. A letter responding to Behman et al. summarizes the issue. If 100 patients are treated according to the current standards, 22 will require surgery for the index SBO, while 19 will have recurrent SBO (16 from conservative treatment, three from operative), totaling 41 surgeries. On the other hand, if those same 100 patients were all operated on (if we accept surgery as the first-line treatment for SBO), 13 patients will experience recurrence, totaling 113 surgeries. In other words, 72 patients would have undergone an unnecessary surgery, needlessly exposing them to the risks of morbidity and mortality associated with any surgery [22].

Limitations

This review has several limitations. Most studies under review were single-center retrospective studies or were confined to a single city or state. This not only limits the diversity of the study population (decreasing the generalizability) but also risks losing study participants to emigration. Much larger studies with a broad and diverse study population (such as Behman et al.) are necessary to begin applying these results to decide on management. Most studies also had a relatively short follow-up period of two to five years. A longer follow-up period is needed to assess the cumulative recurrence risk.

There is a risk of selection bias in the assignment of patients to operative vs. conservative treatment or laparoscopic vs. open surgery. Patients with severe symptoms are likelier to be assigned to operative treatment with open surgery. This negatively skews the results for operative management and open surgery, as severe symptoms imply more severe disease and worse outcomes. Finally, this review is confined to the assessment of recurrence of ASBO only. More comprehensive studies analyzing multiple outcomes are necessary before we can change existing treatment guidelines, but this review provides a starting point.

## Conclusions

The treatment of SBO is a balance between the risks and benefits of conservative and operative management, particularly in the absence of peritonitis or strangulation (major indicators for surgery). Physicians are reluctant to progress to surgery, at least partly due to the “common knowledge” that surgery comes with the risk of recurrence due to the formation of adhesions. This review has proven that popular belief is wrong. This review does not indicate that surgery should replace conservative management as the first-line treatment for ASBO. However, it suggests that we need not delay surgery out of fear of adhesions. Prolonged conservative treatment risks the viability of the bowel, necessitating extensive resection. Shifting to operative management earlier can improve patient outcomes and quality of life.
